# Protein Lipoxidation: Basic Concepts and Emerging Roles

**DOI:** 10.3390/antiox10020295

**Published:** 2021-02-16

**Authors:** Álvaro Viedma-Poyatos, Patricia González-Jiménez, Ophélie Langlois, Idoia Company-Marín, Corinne M. Spickett, Dolores Pérez-Sala

**Affiliations:** 1 Department of Structural and Chemical Biology, Centro de Investigaciones Biológicas Margarita Salas, Consejo Superior de Investigaciones Científicas (C.S.I.C.), 28040 Madrid, Spain; aviedma@cib.csic.es (Á.V.-P.); patricia.gonzalez.jimenez@cib.csic.es (P.G.-J.); 2 College of Health & Life Sciences, Aston University, Aston Triangle, Birmingham B4 7ET, UK; o.langlois@aston.ac.uk (O.L.); i.company-marin@aston.ac.uk (I.C.-M.); c.m.spickett@aston.ac.uk (C.M.S.)

**Keywords:** lipoxidation, electrophilic lipids, oxidative stress, cell signalling, regulation, selectivity, post-translational modifications

## Abstract

Protein lipoxidation is a non-enzymatic post-translational modification that consists of the covalent addition of reactive lipid species to proteins. This occurs under basal conditions but increases in situations associated with oxidative stress. Protein targets for lipoxidation include metabolic and signalling enzymes, cytoskeletal proteins, and transcription factors, among others. There is strong evidence for the involvement of protein lipoxidation in disease, including atherosclerosis, neurodegeneration, and cancer. Nevertheless, the involvement of lipoxidation in cellular regulatory mechanisms is less understood. Here we review basic aspects of protein lipoxidation and discuss several features that could support its role in cell signalling, including its selectivity, reversibility, and possibilities for regulation at the levels of the generation and/or detoxification of reactive lipids. Moreover, given the great structural variety of electrophilic lipid species, protein lipoxidation can contribute to the generation of multiple structurally and functionally diverse protein species. Finally, the nature of the lipoxidised proteins and residues provides a frameshift for a complex interplay with other post-translational modifications, including redox and redox-regulated modifications, such as oxidative modifications and phosphorylation, thus strengthening the importance of detailed knowledge of this process.

## 1. Introduction

Lipids constitute a structurally and functionally heterogeneous group of hydrophobic biomolecules that, among other species, include fatty acids, triacylglycerols, phospholipids, and sterols. Lipids are essential components of cellular membranes, serve as key molecules for the storage of energy, and play important metabolic and signalling functions [1,2]. Lipids can undergo various metabolic transformations, which contribute to their great structural and functional variety. Among these reactions, lipid oxidation is a common transformation that occurs in physiological conditions as a consequence of cellular metabolism but is usually increased under conditions of oxidative stress, i.e., in situations where there is a redox imbalance potentially leading to cellular damage. Both enzymatic and non-enzymatic mechanisms can be involved in lipid oxidation, and may occur by radical or non-radical attack [3]. Oxidized lipids play important roles in inflammation, atherosclerosis, cancer and ageing [4,5,6]. Importantly, some oxidized lipids are or lead to the formation of reactive or electrophilic molecules that can form covalent adducts with other macromolecules, including proteins. Electrophilic lipids can also arise from dehydration or nitration [7,8]. Thus, the term **protein lipoxidation** refers to the non-enzymatic post-translational modification (PTM) of proteins by reactive or electrophilic lipid species, which frequently arise from **lipid oxidation**. There continues to be some confusion in the literature between the terms lipid oxidation and lipoxidation, with some researchers erroneously assuming they are synonymous. In addition, it is important to distinguish both terms from **protein lipidation**. This process refers to the PTM of proteins by lipid moieties, which usually occurs through enzymatic mechanisms, can involve structurally varied lipids, such as glycosylphosphatidylinositol, fatty acids, isoprenoids, and cholesterol, and generally affects protein hydrophobicity and localization and/or protein-membrane or protein-protein interactions [9]. Lipidation can take place at the N- or C-terminus as well as at cysteine, serine, and lysine residues [9,10]. Moreover, lipids can be non-covalently associated with proteins forming complex particles known as **lipoproteins**, which are constituted by a cholesterol-triglyceride core surrounded by phospholipids, other lipids, and embedded proteins [11]. Lipoproteins are essential elements in lipid transport and metabolism, as well as in cardiovascular pathophysiology, and both their lipid and protein components can undergo various oxidations [12]. In this article, we will clarify the terminology (Box 1) and explain the process of **protein lipoxidation**, before addressing advanced aspects of the effects of this PTM and pointing out as yet unanswered questions in the field.

Box 1 **Terminology and Definitions.**

- **Lipid Oxidation**: an overall term encompassing both radical and non-radical (electrophilic) reactions and leading to an increase in the number of oxygens and other heteroatoms (such as nitrogen or chlorine) or a decrease in the hydrogen content of the lipid.
- **Lipid Peroxidation**: a specific form of radical attack, usually at bis-allylic sites in an unsaturated hydrocarbon chain, that leads first to a carbon-centred radical and then the addition of molecular oxygen to form a peroxyl radical (-O-O^•^) on that carbon. The peroxyl radical remains reactive and can abstract hydrogens from adjacent molecules, resulting in a chain reaction and propagation of damage.
- **Lipoxidation**: covalent reaction of reactive and electrophilic lipid products, mostly arising from lipid oxidation, for example, aldehydes or α,β-unsaturated breakdown products such as acrolein and 4-hydrononenal, or cyclopentenone-containing lipids (e.g., 15-deoxy-Δ^12,14^-prostaglandin J_2_) with macromolecules. The targets of lipoxidation include proteins, DNA or head groups of phospholipids.
- **Advanced Lipoxidation End-products (ALEs)**: the covalent adducts formed by the process of lipoxidation.
- **Protein lipoxidation**: the modification of proteins by electrophilic lipids. Although is not an oxidative modification per se, it frequently contributes to the damage to proteins under oxidative stress conditions.
- **Protein lipidation**: enzymatically-catalysed covalent modification of proteins by lipids, which usually enable the proteins to associate with membranes. Typical examples include N-myristoylation, S-palmitoylation, or S-prenylation, as well as the addition of a glycosylphosphatidylinositol anchor.
- **Lipoproteins**: particles formed by amphipathic proteins embedded in a phospholipid monolayer and surrounding an inner core of cholesterol, cholesterol esters and triacylglycerols. They function as lipid transporters and are commonly found in plasma.

## 2. Lipid Oxidation and Protein Lipoxidation

Lipid oxidation can occur enzymatically, catalysed by cyclooxygenases (COX-1/2/3), lipoxygenases (LOX), and cytochrome P450-dependent enzymes (CYP450), or non-enzymatically, when it is mediated by carbon and oxygen-centred radicals [13,14]. Enzymatic pathways give rise to bioactive mediators, such as prostaglandins (PG), thromboxanes and leukotrienes, among others, which have been broadly studied and can act as physiological signalling molecules, with an important role as immunomodulators [15]. Non-enzymatic mechanisms are adventitious oxidations that commonly occur on phospholipids present in cellular membranes and on lipoproteins [12]. Lipids containing unsaturated fatty acyl chains, and particularly those that are polyunsaturated fatty acids (PUFAs), such as linoleic and arachidonic acids, are more vulnerable to attack by different reactive species of oxygen, nitrogen and halogens [16]. In lipid peroxidation, peroxyl radicals are formed as intermediary products [3]. Hence, in a phospholipid bilayer environment, a single radical attack can entail a chain reaction based on a cascade of radical hydrogen abstractions and oxidations. Peroxyl radicals, and to a lesser extent hydroperoxides, are unstable and undergo subsequent reactions including further oxidation, cleavage and cyclization reactions to form a variety of secondary oxidation products (reviewed by [17,18]).

Many of these secondary products, including full-chain length oxidized phospholipids, phospholipids with a truncated fatty acyl chain, and non-esterified breakdown products, are reactive electrophilic products that undergo further rearrangements. Both full-length and shortened fatty acyl chains can contain epoxides, hydroxides, and carboxylic acids as well as reactive carbonyl moieties and α,β-unsaturated alkenal moieties, which are highly reactive [3,19,20,21]. In general, alkenals, and hydroxy- or oxo-alkenals are the most reactive and versatile in terms of their reactivity [17,22,23,24]. Certain reactive lipid products can be formed by enzymatic and/or non-enzymatic reactions. Dehydration of PG synthetised via COX enzymes or non-enzymatically through the isoprostane pathway leads to the generation of cyclopentenone prostaglandins (cyPG) or of keto-PG, which contain unsaturated carbonyl moieties in the cyclopentenone ring and/or in the lateral chains [25,26,27,28]. Lipids can also be attacked by reactive nitrogen species (RNS) to give rise to nitro-alkenals that also can covalently modify proteins [29].

**Figure 1:**
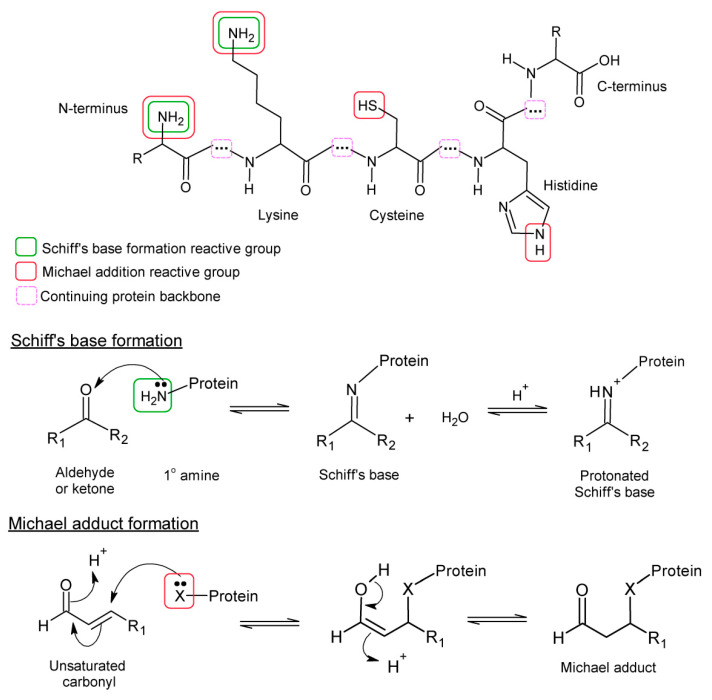
Formation of Schiff’s base and Michael adducts with protein residues. The structures of the lysine, cysteine and histidine residues are shown at the top, with the moieties involved in nucleophilic attack indicated. The histidine imidazole ring exists in 2 resonance forms where the hydrogen can reside on either nitrogen, so either nitrogen can undertake nucleophilic attack. Schiff’s base formation with an amino group is shown in the centre. The Schiff’s base reactions are reversible, involving a hydrolysis reaction. The bottom panel shows Michael adduct formation by nucleophilic group X, where X represents a primary or secondary amine or a thiol. Michael adducts can also decompose by reversal of these reactions, although they are more stable than Schiff’s bases.

Protein lipoxidation involves the formation of Schiff’s bases or Michael adducts. Schiff’s bases are formed by the reaction between carbonyls (aldehydes or ketones) and primary amines, and consequently can only form on lysine or amino-terminal residues in proteins. In contrast, Michael adducts are formed by reaction of a nucleophile with the β-carbon of an α,β-alkenal, and reactivity is enhanced by the presence of an electron-withdrawing group on the γ carbon, such as in 4-hydroxynonenal (HNE) or 4-oxononenal (ONE). In proteins, the nucleophilic group is most commonly cysteine (in the thiolate form), lysine (primary amine in deprotonated form) or histidine (secondary amine in deprotonated form). Adducts with asparagine and glutamine side chains have been reported for certain lipid species despite the lower nucleophilicity of the amino group in an amide [30]. Arginine can form Michael adducts only when deprotonated, which is rare in physiological conditions as the guanidino group is highly basic. This different reactivity of protein residues and lipid species influences the selectivity of protein lipoxidation as will be discussed below. A summary of the mechanisms is given in Figure 1, while detailed reaction mechanisms for the formation of these adducts and their subsequent rearrangements can be found in other reviews [19,31]. Some of the most studied and interesting electrophilic lipids involved in protein lipoxidation are considered briefly below and in Table 1.

Reactive lipid products can be grouped into chemical families according to their reactive chemical groups, which determine their reactivity in lipoxidation reactions. Owing in part to their availability, as well as their biological actions, some reactive lipid products have been much more extensively studied than others. The small, non-esterified aldehydes malondialdehyde (MDA), acrolein, and HNE fall into this category [23,32]. Of these, HNE is the most toxic, acrolein is the most reactive, and MDA is the most mutagenic [33,34,35], reviewed in [10,22,36]; these effects ultimately relate to their potential to cause lipoxidation. In contrast, there are many fewer publications on other aldehydes such as crotonaldehyde, pentanal, hexenal, 4-hydroxy-hexenal (HHE) and 4-hydroxy-dodecadienal, although some of them may be formed physiologically in sufficient amounts to have biological effects and evidence is emerging that they also modify proteins and affect their functions. Substantial research has also been devoted to long-chain species, especially isoprostanes, isolevuglandins, PG species such as cyPG, and nitrated fatty acids (NO_2_-FAs), in part due to their signalling properties [37,38,39,40]. Whereas isoprostanes are important as biomarkers of oxidative stress [41], the behaviour of certain eicosanoids including cyPG, and of NO_2_-FAs as transcription factor agonists and mediators of inflammatory resolution has raised high interest in their potential therapeutic applications. Moreover, cyPG have been used as model compounds for the identification of lipoxidation targets in proteomic studies [27]. Interest in oxidized and nitrated phospholipids as potential agents of lipoxidation is more recent but nevertheless of emerging physiological importance. In summary, the propensity of a lipoxidation adduct to be formed depends on the reactivity of the lipid oxidation product, the nucleophilicity of the target amino acid in the protein, and the stability of the product generated [42]. Furthermore, the initial adducts can undergo additional rearrangements, including reactions with other nucleophilic groups to cause inter- or intra-molecular cross-links, resulting in linear or cyclic stable products [19,43]. Thus, protein lipoxidation contributes to the generation of protein diversity through PTMs, with a variety of structural and functional consequences.

Oxidation of lipid components of membranes occurs in all living organisms. Therefore, the process of protein lipoxidation would be expected to be universal. Indeed, although much work has focused on mammalian and clinical samples, protein lipoxidation has also been studied in plants and microorganisms. For example, immunoblot analysis using monoclonal antibodies against reactive aldehyde-derived protein modifications showed that in spinach leaves grown in normal conditions the oxygen-evolving complex protein 33 was modified by MDA, acrolein and crotonaldehyde [44], while salt stress in *Arabidopsis thaliana* resulted in modification of soluble proteins by HNE, HHE, crotonaldehyde, acrolein and MDA [45]. Excellent reviews that highlight the importance of this PTM in plants are available [46,47]. In contrast, little information on natural lipoxidation and its effects are available for fungi and bacteria, although it has been reported that bactericidal antibiotic treatments lead to the formation of MDA adducts [48]. The chemistry and generic consequences of lipoxidation on protein function are expected to be similar in all of these organisms and will depend on the context and the protein target. However, most of the examples provided in this review will be related to animal models in general and human health in particular, in relation to the pathophysiological consequences of this modification.

**Table 1:** Examples of electrophilic lipid products that can cause lipoxidation.

Reactive Lipid Product	Type	Source	Reactions Reported With	Cross-Linking
Malondialdehyde	bis-aldehyde, isomerizes to β-hydroxy-acrolein	Polyunsaturated chains with ≥3 double bonds	Lys (Michael and Schiff’s) His, Arg, Cys (Michael)	√
Acrolein	Alkenal (3 carbons) (α-β-unsaturated aldehyde)	Polyunsaturated lipids but also other environmental sources	Lys (Michael and Schiff’s) His, Cys (Michael)	√
Crotonaldehyde	Alkenal (4 carbons) (α-β-unsaturated aldehyde)	ω-3 unsaturated lipids (α-linolenic, eicosapentaenoic or docosahexaenoic acid)	Lys (Michael and Schiff’s) His, Cys (Michael)	√
4-hydroxy-2- hexenal (HHE)	4-hydroxy-alkenal (α-β-unsaturated aldehyde)	ω-3 polyunsaturated lipids (α-linolenic, eicosapentaenoic or docosahexaenoic acid)	Lys (Michael and Schiff’s) His, Cys (Michael)	√
4-hydroxy-2-nonenal (HNE)	4-hydroxy-alkenal (α-β-unsaturated aldehyde)	ω-6 polyunsaturated lipids (γ-linolenic or arachidonic acid)	Lys (Michael and Schiff’s) His, Cys (Michael)	√
4-oxo-2-nonenal (ONE)	4-oxo-alkenal (α-β-unsaturated aldehyde)	ω-6 polyunsaturated lipids (γ-linolenic or arachidonic acid)	Lys (Michael and Schiff’s) His, Cys (Michael)	√
15-deoxy-Δ^12,14^-prosta-glandin J_2_ (15d-PGJ_2_)	Cyclopentenone prostaglandin (cyPG)	Arachidonic acid	His, Cys (Michael)	√
15-keto-prostaglandin E_2_	Prostaglandin	Arachidonic acid	Cys (Michael)	X
Palmitoyl-oxovaleroyl phosphatidylcholine (POVPC)	Esterified alkenal	ω-6 polyunsaturated lipids (γ-linolenic or arachidonic acid)	Lys (Michael and Schiff’s) His, Cys (Michael)	X
Palmitoyl-oxononanoyl phosphatidylcholine (PONPC)	Esterified alkenal	ω-6 polyunsaturated lipids (γ-linolenic or arachidonic acid)	Lys (Schiff’s base only)	X
Isolevuglandins (isoLGs) and Isoketals	γ-keto-aldehydes	Arachidonic acid and docosahexenoic acid	Lys (Schiff’s base only)	√
Nitro-oleate and nitro-linoleate	Nitro-fatty acids (NO_2_-FAs) (can be esterified in PLs)	Unsaturated fatty acyl chains (e.g., oleoyl or linoleoyl)	Lys, His, Cys (Michael) (nitro-alkylation)	X
Chloro-hexadecanal or chloro-octadecanal	Chloro-fatty aldehydes	Plasmenyl phospholipids (palmitate or stearate attached by vinyl ether bond)	Lys (Schiff’s base only)	X

Diverse electrophilic lipid species from different origins can covalently bind to proteins (protein lipoxidation) through the formation of Michael and/or Schiff’s adducts with nucleophilic residues. Some of the reported species can induce protein crosslinking. For detailed information, please see [23,24,49,50,51].

## 3. Functional Consequences of Lipoxidation

**Figure 2:**
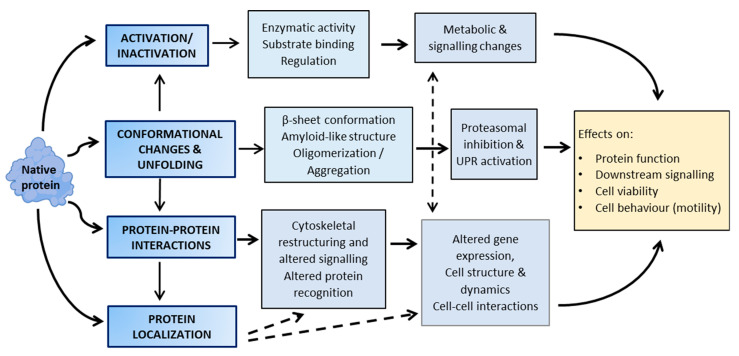
Overview of the biochemical effects of protein lipoxidation, which are highly interrelated.

Proteins serve a wide array of functions in the cellular and extracellular contexts, which are subjected to complex control, often involving enzymatically generated PTMs, such as phosphorylation, glycosylation, ubiquitination, methylation and lipidation, to mention just a few. Non-enzymatic modifications of proteins also involve a plethora of chemical transformations of protein residues that, especially at high levels, can have deleterious effects on protein structure and function. Nevertheless, oxidative and electrophilic PTMs, including lipoxidation, may also contribute to the regulation of protein function and play an important role in redox signalling [19,52,53]. Below, the different effects of lipoxidation of proteins are considered, together with their potential as physiological regulatory processes. Table 2 provides examples of protein targets of lipoxidation and of its functional consequences, which are schematically illustrated in Figure 2.

Lipoxidation of residues located at or near the active site of enzymes can bring about changes in enzymatic activity, for example through alterations of their active conformation or by blocking the binding of substrates [54]. Lipoxidation-induced enzyme inactivation has been reported for aldehyde dehydrogenase (ALDH2) [55] and pyruvate kinase [33], and may simply represent damage. In contrast, both activation or inactivation have been documented for aldo-ketoreductase B1 (AKR1B1), depending on the size of the electrophilic moiety causing the adduct [56,57]. As well as reacting with metabolic enzymes, electrophilic lipids can target proteins and enzymes involved in signal transduction, such as the phosphatases phosphatidylinositol 3,4,5-trisphosphate 3-phosphatase (also known as phosphatase and tensin homolog PTEN) and protein phosphatase 2 (PP2A). PTEN can be modified by acrolein, HNE, prostaglandin A_2_ (PGA_2_) or 15-deoxy-Δ^12,14^-prostaglandin J_2_ (15d-PGJ_2_) [58,59], whereas PP2A has been recently reported to be modified by HNE [60], resulting in both cases in inhibition, which indirectly affects the phosphorylation status of their targets and therefore, their downstream signalling pathways. Certain histone deacetylases (HDACs) can also be inhibited by HNE and 15d-PGJ_2_, which affects gene expression [61]. In contrast, activation of metalloprotease-9 by acrolein has been reported [62], with potential implications for tissue damage in a variety of inflammatory conditions.

Electrophilic lipids can also induce protein conformational changes, which may affect activity indirectly, lead to partial unfolding or alter protein-protein interactions. Conformational changes often cause changes in secondary structure, such as an unfolding or increase in β-sheet content, which tend to favour the formation of amyloid-like structures and aggregation of proteins [63]. Examples of specific proteins undergoing these changes upon lipoxidation are the ubiquitin hydrolase ubiquitin carboxy-terminal hydrolase L1 (UCH-L1), which is present in neurofibrillary tangles or Lewy bodies in Parkinson disease [64], and glutathione-S-transferase (GST), which is cross-linked in the presence of 15d-PGJ_2_ [65]. Moreover, an increased immunoreactivity with anti-ALE antibodies has been observed in a number of protein aggregates associated with pathophysiological conditions, including β2-microglobulin amyloid deposits associated with uremic complications [66]. This suggests a role for lipoxidation in the pathophysiology of these conditions. Although lipoxidation is more likely to affect nucleophilic residues located at the protein surface, small aldehydes can gain access into protein folds or binding pockets, leading to protein instability and unfolding. This can increase the exposure of hidden residues, rendering the protein more vulnerable to further modification [31]. As a result, the unfolded protein response (UPR) may be activated [67,68,69,70]. In addition, cross-linking or aggregation of proteins prevents their degradation via the 20S proteasome; inhibition of proteasome function may then occur, which directly affects cell viability and commonly results in cell death [71,72].

**Table 2:** Examples of lipoxidation targets.

Category	Protein	Lipid	Residue	Implication	Reference
Cytoskeletal protein	Vimentin	15d-PGJ_2_, PGA_1_ HNE	Cys328	Filament reorganisation	[73,74] [75,76]
	GFAP	15d-PGJ_2_, PGA_1_	Cys294	Filament reorganisation	[77]
	Actin	HNE PGA_1_ 15d-PGJ_2_ Acrolein	Cys374 Cys374 Cys374, His87, His173	Electrophilic scavenger, filament disruption	[78] [79] [80] [81]
	Tubulin	HNE	Cys295	Filament reorganisation	[75]
Metabolic enzymes	AKR1B1 AKR1B AKR1B10	Acrolein HNE PGA_1_	Cys298 Cys298 Cys299	Activation Inhibition Inhibition	[56] [57] [82]
	α-Enolase	HNE 15d-PGJ_2_	? *	Inhibition	[83] [80]
	Soluble epoxide hydrolase	15d-PGJ_2_	Cys521	Inhibition	[84]
	Pyruvate kinase	15d-PGJ_2_ Acrolein, HHE, MDA HNE, ONE	? Cys152, Cys358, Cys423, Cys474 Cys424, His439	? Inhibition Inhibition	[74] [33] [85]
	Pin1	HNE	Cys113	Inhibition	[86]
Chaperones	Hsp 90	15d-PGJ_2_ PGA_1_ HNE, ONE	? Cys572	Inhibition	[74] [73,87] [88]
	Hsp 70	cyPG HNE	? Cys267	Inhibition	[84,87] [89]
Transcription factor	PPARγ	NO_2_-FAs 15d-PGJ_2_	Cys285 Cys285	Activation	[90] [91,92]
	P53	15d-PGJ_2_	Cys277	Inhibition	[93]
	NF-κB	15d-PGJ_2_, PGA_1_	Cys38 (p65) and Cys62 (p50)	Inhibition	[94,95]
	STAT3	15-keto-PGE_2_	Cys259	Inhibition	[96]
	AP-1	15d-PGJ_2_	Cys269 (c-Jun)	Inhibition	[97]
Membrane receptor	Estrogen receptor α	15d-PGJ_2_	Cys227, Cys240	Inhibition	[98]
	EGFR	HNE	?	Activation (low levels); inhibition (high levels)	[99]
	TRPA	15d-PGJ_2_	Cys421, Cys621	Activation	[100]
Regulatory proteins	Keap1	NO_2_-FAs HNE 15d-PGJ_2_	Cys151, 273, 288 Cys 273, Cys288	Inhibition	[101,102] [103] [101,102,103,104]
	IKK	HNE cyPG	? Cys179	Inhibition	[105] [106]
	H-Ras	15d-PGJ_2_, PGA_1_	Cys118, Cys181, Cys184	Activation	[107,108]
Signalling protein	PTEN	Acrolein, HNE, PGA_2_, 15d-PGJ_2_	Cys71, Lys327	Inhibition	[58,109]
	Akt	HNE	His196, His267, Cys311	Inhibition	[110]
	PP2A	HNE	?	Inhibition	[60]
Epigenetic regulation	Sirt2	Acrolein, HNE	Cys482	Inhibition	[111]
	HDACs	Acrolein HNE, 15d-PGJ_2_	Cys274 Cys 274	Inhibition Inhibition	[112] [61]
Mitochondrial proteins	DRP1	15d-PGJ_2_	At least Cys644	Fission inhibition	[113]
	Cytochrome c	HNE	His196, His267, Lys87	In vitro modification	[114]
	Aconitase	HNE	Cys99, Cys358, Cys421, Cys424, Cys565	Inhibition	[115]
Others	Albumin	Δ^12^-PGJ_2_ HNE, acrolein	His146 Cys34	?	[116] [117]

* ? indicates that either the site of adduction or the biological effect were not determined in this study.

In some cases, conformational changes induced by binding of electrophilic lipids cause protein activation, either through direct or indirect mechanisms. The transcription factor peroxisome proliferator-activated receptor γ (PPARγ), which is redox-sensitive [90], illustrates this point nicely. Conformational changes in PPARγ during its activation trigger heterodimerization with retinoid X receptor, thus promoting the induction of anti-inflammatory genes and repression of pro-inflammatory genes such as nitric oxide synthase (iNOS) [118,119]. Several electrophilic lipids, including NO_2_-FAs, oxo-octadecadienoic acid (oxo-ODE) and cyPG cause covalent modification of PPARγ by Michael addition at the Cys285 residue and promote conformational changes necessary for its full activation [90,91,92,120]. Another example is the nuclear factor erythroid 2–related factor 2 (Nrf2), a transcription factor that is central in the antioxidant response and is indirectly activated by lipoxidation. Normally, Nrf2 is bound to its regulator Keap-1, an adaptor for ubiquitination that enables Nrf2 proteasomal degradation under non-stressed conditions. Modification of critical cysteines in Keap-1 causes a conformational change disrupting the degradation machinery and allowing nuclear translocation of newly synthetized Nrf2 and activation of its target genes [121,122]. Therefore, lipoxidation-induced conformational changes can also affect protein–protein interactions. The assembly of cytoskeletal proteins provides clear examples. Lipoxidation of a single cysteine residue (Cys328) in the intermediate filament protein vimentin can alter filament morphology or cause a complete disruption of assembly with the formation of aggregates [123]. Similarly, lipoxidation of actin at Cys374 can disrupt microfilaments if it occurs in a substantial proportion of molecules [80]. Lipoxidation of tubulin alters the morphology of microtubules, inducing a thinner appearance [74]. Some of these alterations could be due to steric hindrance caused by the addition of bulky moieties. In addition, changes in protein charge due to lipoxidation can also affect protein-protein interactions as reported for the binding of lipoxidised albumin to the receptor of advanced glycation end products (RAGE) [124]. Finally, lipoxidation can alter protein–DNA interactions, as is the case for transcription factor NF-κB, which is responsible for the signalling cascade that controls the expression of many proinflammatory genes. Direct lipoxidation of subunit p65 (Cys38) or p50 (Cys62) by 15d-PGJ_2_ or PGA_1_ has been reported to inhibit NF-κB binding to the DNA [94,95], thus reducing expression of proinflammatory genes.

As mentioned above, lipoxidation can influence protein subcellular localization indirectly through changes in protein interactions or degradation. However, the addition of electrophilic lipid moieties can also alter membrane targeting, either directly by the action of the bound lipid or indirectly if lipoxidation occurs on residues or domains involved in subcellular targeting or alters the transport mechanisms. Lipoxidation could increase the hydrophobicity of the molecule by altering its charge or introducing acyl groups, which could mimic the effects of lipidation and thus influence membrane interaction. The protein H-Ras poses an interesting example because it can be modified by cyPG at Cys181 and Cys184 residues [107,108], which are sites of palmitoylation and therefore important for subcellular targeting. Indeed, modification of these residues in H-Ras by different moieties has been shown to correlate with its localization to the plasma membrane or endomembranes [125]. In turn, lipoxidation of glyceraldehyde-3-phosphate dehydrogenase (GAPDH), although it inactivates the enzyme, induces its translocation to the nucleus where it is involved in the induction of apoptosis [62]. Interestingly, lipoxidation of Chromosomal Maintenance 1 (CRM1) inhibits nuclear protein export [126], therefore inducing nuclear accumulation of its substrates.

Although this review is more focused on lipoxidation in the cellular context, protein lipoxidation in the extracellular milieu and the bloodstream has important consequences, including increased immunogenicity, transfer of proinflammatory and damage signals and contribution to a variety of pathophysiological processes [12,127]. In summary, lipoxidation can impact essential processes including cell signalling and metabolism, cytoskeletal function, protein degradation and gene expression. Moreover, regulation of these processes by lipoxidation is often double-sided, with either protective or deleterious effects depending on the protein target, the nature and the levels of the electrophilic lipid species and cellular context factors, which will be discussed below.

## 4. Selectivity and Protein Targets of Lipoxidation

Investigations of reactive oxidized lipid-protein adducts on entire proteomes have shown that not all proteins of a proteome are subject to lipoxidation [75,87,128], thus suggesting that this process is both site-specific and protein selective. Protein lipoxidation appears to occur on specific sets of proteins within the cellular proteome, which act as “hot spots”. In the circulation, albumin seems to be very susceptible to lipoxidation because of its abundance and of the high reactivity and accessibility of some nucleophilic residues (Cys34 and Lys199) [129]. In the cellular environment, the chaperones Hsp70 and Hsp90, Keap1, and the cytoskeletal proteins tubulin, actin and vimentin are frequent targets of lipoxidation [74,130]. Also, adducts seem to be more common in the cytosol and nucleoplasm than in the membrane, although this may depend on the type of lipid and on the difficulties to analyse membrane proteins [73,131,132,133]. In addition, certain cellular pathways, such as defence responses, or subcellular localizations appear particularly susceptible. Studies on the mitochondrial proteome showed that respiratory chain and tricarboxylic acid cycle (TCA) proteins, as well as transporters, are the most represented proteins undergoing lipoxidation [134,135]. Codreanu et al. identified HNE and ONE protein adducts in THP-1 and RKO cell lines and performed a Gene Ontology (GO) analysis, which showed that their function was predominantly involved in folding, RNA metabolic and glucose catabolic processes, cytoskeletal regulation and protein synthesis and turnover [136]. This is in agreement with previous studies that identified proteins related to the cytoskeleton, stress and immune responses, metabolic processes and glycolysis, regulation of translation and RNA binding as targets for HNE or cyPG in various cellular models [74,75,87]. Table 2 gives also examples of the site-specificity of lipoxidation on some target proteins, as determined in studies performed mostly in vivo or in cellulo, using physiological or pathophysiological treatment levels of electrophilic lipids and employing mutagenesis approaches to investigate the biological effect. Interestingly, information on sites of modification has also been obtained from in vitro studies, which have provided fundamental information on relative residue susceptibility and functional consequences, although in some cases yielded a higher number of modified residues. Some examples are shown in Table 3.

Why are some proteins more susceptible to lipoxidation than others? Some of the proteins mentioned above (albumin, chaperones, cytoskeletal and glycolytic proteins) are highly abundant in cells; as chemical reactions are concentration-dependent, there is a higher probability that abundant proteins will be both modified and detected during the analysis. However, this is not always the explanation, as illustrated by the lipoxidation of transcription factors and signalling proteins, which are minor cellular components. Instead, the biochemical characteristics of the protein or enzyme come into play. An important factor is the reactivity of amino acid sidechains by Schiff’s base formation or Michael addition, which is determined by their nucleophilicity [24,141]. Generally, the high nucleophilicity of the cysteine thiol makes it more reactive than the imidazole in histidine or amino group in lysine [134]. This agrees with the observation that out of 398 residue sites targeted by HNE in HEK293T cells, most (85.9%) were cysteines (342 residues), and only 27 were histidines (6.8%) and 29 lysines (7.3%) [142]. Moreover, in proteins with multiple cysteine residues frequently only one or two of them are targets for lipoxidation. For instance, Cys34 of albumin and Cys374 of actin are the most reactive and commonly modified residues of these two proteins [129], while the cysteine residues located in the C-terminal segments of several proteins of the Ras superfamily, including H- and N-Ras and Rac1, are lipoxidised [107,143]. This selectivity can arise because of a low pK_a_ of the cysteine, which is influenced by its chemical microenvironment; the proximity of basic amino acids, such as positively-charged lysines, a metal centre, a catalytic triad or aromatic amino acids, lower the pK_a_ and favour the formation of the more nucleophilic thiolate form, which is more prone to oxidation and lipoxidation [144,145,146,147,148]. Consequently, those thiols can act as redox sensors because they are highly responsive to various oxidative modifications. Examples of proteins with unusually low cysteine pK_a_s include protein tyrosine phosphatases, thioredoxin (Trx) and peroxiredoxins (Prx). Lysine and histidine sidechains are commonly positively charged at physiological pH, but their pK_a_s can also be modulated by their local environment through hydrogen bonding and charge stabilization, though as yet this has been less studied.

Another factor important in determining target residues in a protein is their solvent accessibility. A meta-analysis of human proteins identified as targets of HNE and acrolein modification showed that adducted residues were, on average, more accessible than the unreactive ones [141]. Similar findings were reported for the modification of pyruvate kinase by 3 small aldehydes [33]. The influence of nucleophilic residue accessibility was studied in the context of the modification of mitochondrial proteins by endogenous 2-alkenals [134], and it was found that local flexibility (B-factor values) and solvent accessibility areas were generally higher on 4 out of 5 cysteine residues that were found adducted on mitochondrial malate dehydrogenase. Interestingly, it has been reported that adducted residues are surrounded by a greater number of aromatic residues and fewer aliphatic residues than unreactive nucleophile residues [141].

Clearly, the nature and concentration of the electrophilic lipid species also determine the nucleophilic side chains targets in proteins, as explained above [33,42,115] and illustrated by the information in Table 2. There is good evidence that size and structure play an important role in the selectivity of protein modification. A study on cultured fibroblasts found that the closely related cyPG PGA_1_ and 15d-PGJ_2_ modified distinct and not totally overlapping subsets of proteins, with some targets clearly being preferentially modified by one of the cyPG [82,149]. Molecular simulations and docking studies have provided insight into the structural basis of the interaction between electrophilic lipids and proteins, documenting the basis for selectivity. PGA_1_ undergoes interactions with residues at the active site of AKR1B1 or B10, which favour the formation of a Michael adduct [82]. Similarly, favourable interactions have been proposed for the addition of 15-keto-PGE_2_ to signal transducer and activator of transcription 3 (STAT3) [96].

**Figure 3:**
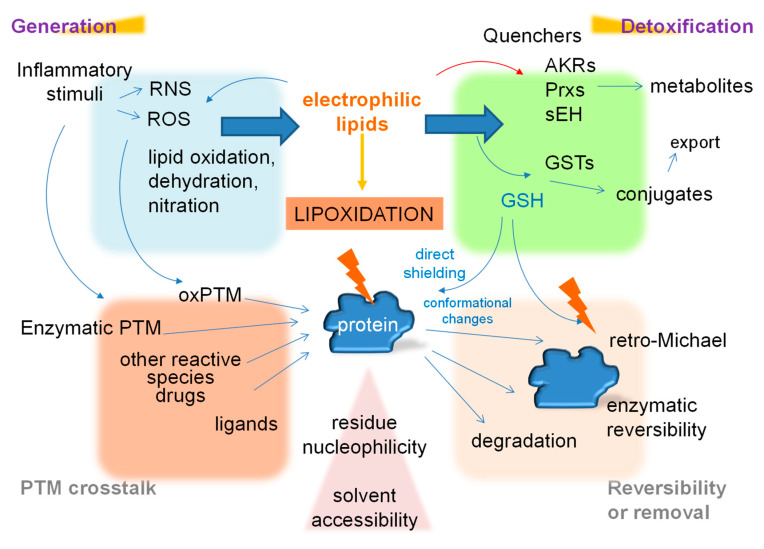
Scheme showing the main factors affecting lipoxidation, from the generation of reactive species and PTM crosstalk on the left to mechanisms of detoxification and reversibility on the right. Please, see text for details and abbreviations.

Overall, it can be seen that lipoxidation “hot spots” occurring at the level of the proteome and the protein are highly interdependent, and influenced both by the nature of the protein, the electrophilic lipid and their concentrations. The situation is further complicated by the fact that other aspects of the cellular environment can influence properties of the electrophilic lipids or the adducts, including their availability and stability, thus impacting on the selectivity of the modification. This is considered further in the next sections and illustrated in Figure 3.

## 5. The Emerging Role of Lipoxidation in Cellular Regulation

PTMs involved in signalling are generally considered to be fast, reversible and specific, for these properties provide a high degree of control over the downstream processes. As a non-enzymatic modification, the role of protein lipoxidation as a signalling mechanism has been controversial, due in part to an incomplete understanding of the possibilities of regulation of this process. Nevertheless, protein lipoxidation can be modulated at several levels, including at the level of generation of the reactive species or their precursors, through their metabolism/detoxification, which influences their availability, or at the level of the stability of adducts, which can be regulated by adduct reversal. Some electrophilic lipids, including cyPG, derive from the dehydration of PG, which in turn are synthesized by regulated enzymatic processes that can be induced under situations of inflammation. Knockout of key enzymes involved in cyPG generation, such as PGD synthase, results in a decreased production and action of these electrophilic lipids [150]. Consequently, inhibitors of the phospholipases, COX and/or PG synthases involved in the enzymatic steps of PG synthesis may result in a reduction of the generation of the electrophilic lipids derived from them [151,152].

The metabolism or detoxification of reactive lipids or their precursors can be catalysed by diverse enzymes, thus influencing their availability and therefore the extent of lipoxidation. GSTs constitute a well-characterized family of enzymes that catalyse the conjugation of reduced glutathione (GSH) to electrophilic lipids to generate more soluble species that can be exported by multidrug resistance transporters, thus reducing their cellular availability [153,154,155,156]. Several electrophilic lipids, including cyPG and HNE are substrates of GST [153,154,156,157], for which enzymatic and non-enzymatic conjugation GSH has been shown to decrease their levels and activity [153,156].

Other enzymes that have been proposed as mediators of lipid detoxification include soluble epoxide hydrolase (sEH), which can metabolise epoxy fatty acids (PUFAs) [158], phospholipid hydroperoxide glutathione peroxidase and the Prxs [29]. A wide and diverse group of enzymes can detoxify aldehyde-containing electrophilic lipids. For instance, several isoforms of the aldo-keto reductase (AKR) family use NAD(P)H to reduce aldehyde groups of some electrophilic lipids such as acrolein, HNE or cyPG precursors [159,160], thus decreasing their availability and biological effects. Other enzymes that can reduce the aldehyde group of HNE, including aldose/aldehyde reductase (ALR), alcohol dehydrogenase (ADH), aldehyde dehydrogenase (ALDH), alkenal reductase (AER), alkenal hydrogenase (ALH), and alkenal/one reductase (ACR) have been reported to reduce its bioavailability and reactivity in both plants and humans [32,46]. Thus HNE detoxification can occur both by conjugation with GSH or direct detoxification by ADH or ALDH [32,161]. Importantly, several enzymes involved in detoxification of electrophilic lipids, including GST, AKR and soluble epoxide hydrolase are targets for reactive lipids themselves, which increases the complexity of these interactions [65,82,84].

A key feature of mechanisms considered to participate in cell signalling is that they need to be reversible, either directly or indirectly; lipoxidation shows potential reversibility through several mechanisms. Although both Schiff’s and Michael adducts are chemically reversible, Schiff’s adducts are more labile and reversal can occur spontaneously in aqueous solution [31], whereas Michael adducts are in general more stable. However, retro-Michael reactions are also possible under some circumstances. An adduct formed between AKR1B1 enzyme and a biotinylated analogue of PGA_1_ is partially reversed by incubation in the presence of an excess GSH in vitro [162]. Furthermore, Michael adducts generated by HNE and ONE can be reverted in vitro and in cells as demonstrated by quantitative chemoproteomic analysis [163] and kinetic studies [164]. In cells, the involvement of enzymatic mechanisms in the reversal of lipoxidation has been proposed. Acrolein protein adducts are reversed in bronchiolar epithelial cells by mechanisms dependent on GSH and Trx 1 [165]. In addition, the deacetylase Sirt2 has been reported to catalyse the enzymatic reversion of acrolein lipid adducts [166,167], as revealed by quantitative analysis [163]. NO_2_-FAs are a special case, since their adducts with cysteine residues are reversible, and there is consensus that they can behave as signalling mediators [168,169], although there is still much to be learned about the potential regulation of their generation and site of action.

The functional reversal of lipoxidation can also be achieved indirectly by degradation of the lipoxidised proteins and substitution by newly synthesized proteins with the consequence of the recovery of the biological effect [50,170]. As stated above, lipoxidation is frequently associated with inhibition of proteasomal degradation. Therefore, removal of protein-lipid adducts has been proposed to occur through lysosomal degradation and autophagy [171], especially of those containing α,β-unsaturated carbonyl groups or aldehydes.

Specificity is an important aspect of regulatory processes. Besides the selective aspects of lipoxidation discussed above, several lines of evidence indicate that in some cases protein lipoxidation can display distinct structure-function relationships. Lipoxidation of members of the AKR family by different species can lead to distinct functional consequences. Whereas modification by small moieties such as acrolein at Cys298 of AKR1B1 increases its catalytic activity [56], the addition of bigger reactive lipids such as HNE or certain cyPG promotes the inactivation of these enzymes [57,162]. The assembly of cytoskeletal protein vimentin is also sensitive to modulation by lipoxidation of its single cysteine residue and displays differential features depending on the structure of the adducted lipid [123]. However, whether this is a physiological mechanism of signalling is not known at present.

## 6. The Dependence of Lipoxidation on the Cellular Environment

Reactive lipids can exert both beneficial and detrimental effects on the cell [38]. Which of these predominates depends on many factors that influence the generation and final fate of the lipids and their adducts, including the cell type, the status of the antioxidant defences, and the concurrence of other reactive species or stimuli. The complexity of this balance is illustrated by the fact that electrophilic lipids can induce the expression of antioxidant defence enzymes, but at the same time influence their activity either directly, through lipoxidation, or indirectly, by triggering the production of reactive oxygen species (ROS) (see [172] for review).

The antioxidant system of the cell includes both enzymatic and non-enzymatic elements. Enzymes contributing to the antioxidant defence include the superoxide dismutase (SOD), heme oxygenase-1 (HO-1), catalases, glutathione peroxidase, Prxs, glutathione reductases and Trx. Small-molecule antioxidants include GSH, vitamins, lipoic acid and several cations such as Mn, Fe, Cu or Zn [63,173]. An example of the interplay between these factors and lipoxidation is provided by the cell- and species-dependent subcellular compartmentalization of cyPG accumulation and effects. For instance, differences in the main site of inhibition of the NF-κB activation pathway (through either cytoplasmic or nuclear events) have been attributed to the distinct subcellular distribution and abundance of antioxidant defences, i.e., GST activity and GSH content, in different cell types (see [172] for review). Therefore, increased levels of GSH, or enzymes involved in cyPG metabolism such as GST, are associated with lower levels of cyPG modification, and vice versa. Moreover, the stability of the adducts between electrophilic lipids and GSH depends on the lipid species [174], for which GSH levels will not affect the availability of electrophilic lipids uniformly. In addition, the observation that non-hydrolysable GSH analogues protect certain proteins, e.g., GSTp, from lipoxidation suggests the involvement of steric effects or induction of conformational changes in the protective effects of GSH [65]. Finally, these elements are dynamic, which increases the complexity of these interactions. For instance, cytosolic GSTs can translocate to the nucleus, altering the location of protection [175,176].

The complexity of these interactions is even higher since electrophilic lipids also influence the activity of the detoxifying enzymes. Certain electrophilic lipids can bind and inactivate GST and/or induce its crosslinking [65,177]. In addition, the reduced form of Prx is a direct target of HNE [178] whereas Trx can be modified by acrolein and HNE at the non-catalytic Cys73 [179] and by cyPG at Cys35 and Cys69 [180]. Moreover, TrxR is also a target for lipoxidation [181]. In most cases, lipoxidation is associated with inhibition of these targets, thus inducing the accumulation of cellular ROS. Nevertheless, as stated above, interaction with GSH can protect these enzymes from lipoxidation.

Vitamins may act as both pro- and anti-oxidants and their interactions with electrophilic lipids and lipoxidation appear to be complex and dependent on the experimental system. Examples of these interactions include reports on vitamin E decreasing lipid peroxidation in clinical trials or studies [182] and the ability of vitamin B6 to sequester intermediates of lipid peroxidation and reduce the formation of lipoxidation adducts [183,184]. Nevertheless, some actions of vitamins are controversial and the reader is referred to specialized reviews on this topic [170,173,185].

Divalent cations such as iron, copper, zinc or manganese also influence the redox state of the cell through various mechanisms including radical generation through the Fenton reaction (iron and copper), radical scavenging (manganese) or acting as cofactors for antioxidant enzymes (reviewed in [173]). In the context of lipoxidation, **zinc** presents special interest. Zinc competes with iron and copper in their coordination environments and suppresses their redox activity in Fenton chemistry. Interestingly, Zn^2+^ can interact with the thiolate group of cysteine, with important implications in Redox Biol, and the imidazole group of histidine [186], both of which are strong nucleophiles and frequent targets of lipoxidation. Zinc binding can affect the reactivity of cysteine residues and/or protect them from chemical modification, including lipoxidation [187,188]. The cytoskeletal protein vimentin provides an example of this protection both in vitro and in cells, since zinc availability in the physiological range protects the single cysteine residue of vimentin from alkylation, oxidation or lipoxidation in vitro, and preserves the integrity of the network in cells [188]. In turn, oxidation or lipoxidation of cysteine residues involved in the interaction with zinc releases this metal and contributes to zinc toxicity in cells [189]. On the other hand, metal-ion chelators inhibit lipoxidation reactions through the elimination of metal ions [170]. Some examples of compounds which can act as metal-ion chelators include citric acid (relatively non-specific chelator) [170], polyphenols and flavonoids [173].

Among other factors related to the cellular or extracellular context that can modulate lipoxidation is the presence of scavengers or quenchers. While the two terms are often used interchangeably, scavengers could be considered non-covalent binders of electrophilic lipids, whereas quenchers would be strong nucleophilic compounds reacting with the electrophilic derivatives leading to unreactive products. Thus, scavenging or quenching of electrophilic lipids could prevent protein lipoxidation. Therefore, in addition to endogenous compounds entailing this activity, exogenous natural and synthetic quenchers are being studied as potential therapeutic tools [170,190]. One of the best-studied examples is the dipeptide carnosine composed of β-alanine and histidine, which has served as the basis for the synthesis of more stable analogues, one which, known as carnosinol, has been found to decrease lipoxidation and showed beneficial effects in animal models of disease [191].

Finally, the presence of other reactive species, either endogenous or exogenous, such as drugs and their metabolites can influence lipoxidation by causing alterations in the cellular antioxidant systems or the protein targets, and even compete for target residues contributing to PTMs crosstalk. Therefore, factors from the cellular context may influence the extent and the site of protein lipoxidation, contributing to its selectivity and accounting for potential differences in the results from in vitro and in in vivo studies.

## 7. Interplay among Post-Translational Modifications

Lipoxidation can induce oxidative stress, thus eliciting the formation of further reactive species, responsible for additional PTMs leading to chain reactions with implications in different cellular processes [192]. Moreover, lipoxidation of enzymes involved in PTMs, such as phosphatases, kinases or deacetylases (see above), can affect PTMs. Therefore, a complex interplay between PTMs can take place involving lipoxidation, modifications by other reactive species, and activation or inhibition of proteins catalysing other PTMs. Moreover, direct cooperation or competition among PTMs can occur on the same proteins or residues, which could result in an increase of protection from lipoxidation, thus contributing to the generation of highly diverse proteoforms and the complexity of events determining the overall outcome.

Among reactive species potentially competing with electrophilic lipids for modification of proteins are species derived from the oxidation of sugars, ROS and RNS and other small molecules, like metabolites of certain amino acids, or even drugs. The modification of cysteine residues can provide numerous examples of this potential competition, given their capacity to accommodate multiple modifications [193,194]. In general, it could be considered that cysteine oxidation in its various forms, including formation of disulphide bonds, sulfenic and sulfonic acids, nitrosation, etc., would make the residue less available for lipoxidation. Nevertheless, sulfenic acids have been reported to be more reactive towards certain electrophilic compounds [195], while some disulfides are highly reactive with oxidants [196]. Therefore, in certain cases, cysteine reversible modifications, including disulphide formation, glutathionylation, nitrosation, or addition of NO_2_-FAs, could confer protection against more deleterious ones involving the formation of stable adducts, protein crosslinks, unfolding or aggregation [197,198].

Several previously discussed lipoxidation targets provide examples of these protective mechanisms. The enzyme AKR1B1 possesses seven cysteine residues, two of which, Cys298 and 303, are close to the active site. Formation of a disulphide bond between these cysteine residues reversibly inactivates the enzyme. Nevertheless, this modification could prevent a more stable modification causing either activation or inactivation of the enzyme. For instance, the cyPG PGA_1_ forms an adduct with Cys298 resulting in inhibition. The single cysteine residue of vimentin, Cys328 can also be the target for a wide variety of modifications. Reversible modifications of this residue include disulphide formation, nitrosation or glutathionylation, which can have different functional consequences [199]. Nitrosation, in particular, appears to elicit only minor alterations of vimentin assembly in vitro [200]. Therefore, it would be interesting to explore whether this reversible modification can play a protective role against more disruptive modifications such as CyPG addition. Interestingly, in vitro incubation of vimentin or a PPARγ construct with the nitrated phospholipid 1-palmitoyl-2-oleyl-phosphatidylcholine (NO_2_-POPC) shields their cysteine residues from alkylation [201]. Whether this is due to the occurrence of competing modifications requires further study.

Lipoxidation maintains an important interplay with phosphorylation through various mechanisms. As briefly discussed above, several kinases and phosphatases contain reactive thiols that are subjected to redox control and can be targets for several electrophilic species. Examples of kinases with reactive thiols include protein kinase A (PKA), PKG, PKC and Ca^2+^/calmodulin-dependent protein kinase II (CaMKII) [202,203]. Moreover, both 5′ AMP-activated kinase and AKT have been shown to be direct targets for lipoxidation by HNE [110,204]. Moreover, kinase cascades can be indirectly activated by lipoxidation. Monomeric GST binds and sequesters several stress kinases such as c-Jun N-terminal kinase (JNK) or Traf-2 or binds to their substrates [205,206] in such a way that oxidation or lipoxidation-induced GST crosslinking results in the activation of the corresponding stress signalling pathways [65,205,207]. In turn, lipoxidation of Ras proteins elicits their activation and that of downstream kinase cascades, including MAPKs, phosphoinositide 3-kinase (PI3K) and AKT [107,208].

In addition, several serine and tyrosine phosphatases can be regulated by redox mechanisms and are targets for lipoxidation, which can result in activation or inactivation of phosphatase activity, generally leading to reciprocal changes in the phosphorylation level of its substrates and modulation of the corresponding pathways [209]. Examples of phosphatases subjected to this control are PP2B, PP1, PP2A and PTEN. Lipoxidation of PP2A by PGA_1_ through the formation with a Michael adduct at Cys377 reduces the phosphorylation state of Tau [210]. In contrast, several electrophilic lipids, including acrolein, HNE and cyPG covalent modify and inactivate PTEN, resulting in activation of the AKT pathway and increased proliferation in several cancer cell lines [58,59]. Recently, the formation of an adduct of 15d-PGJ_2_ with Cys136 of PTEN has been reported [211]. Importantly, the possibility that electrophilic lipids can alter the expression levels of kinases or phosphatases provides an additional layer for interplay [212]. Therefore, alterations in protein phosphorylation status could be commonly associated with lipoxidation, and the occurrence of both modifications on the same target would influence the final outcome. For instance, in the case of vimentin, lipoxidation and phosphorylation appear to cooperate to induce filament disassembly [123]. However, in the case AKT, HNE indirectly promotes its phosphorylation, which would normally lead to activation but at the same time, directly modifies the enzyme, resulting in inhibition [110].

Importantly, direct competition between lipoxidation and phosphorylation could occur at histidine residues, which can be targets for both kinds of modification [213], although this potential interplay, to the best of our knowledge, has not been explored in detail. Other unusually phosphorylated amino acids include lysine and arginine.

Lipoxidation can also affect protein acetylation. Several HDACs are targets for lipoxidation. Indeed, a feedback mechanism controlling the expression of stress genes has been proposed that depends on the modification of certain HDACs by cyPG and HNE [61]. Moreover, lipoxidation of Sirt3 by HNE associates with mitochondrial protein hyperacetylation [214]. Notably, as lysine residues are targets for both lipoxidation and acetylation, the interplay between both modifications could occur also at this level. Similar interactions that could affect other modifications such as lysine ubiquitination or formylation deserve investigation.

## 8. Conclusions

In summary, modification of proteins by lipoxidation can elicit varied functional consequences and affect a myriad of intracellular processes. Being a non-enzymatic modification, envisaging potential regulatory roles of lipoxidation is controversial. The chain reaction provoked by lipid oxidation could expand in a flood-like manner affecting multiple proteins and pathways. Nevertheless, accumulating evidence indicates that protein lipoxidation is not a random process, which could be subjected to regulation at several levels. Indeed, low or moderate level protein lipoxidation appears to be involved in cellular defence responses and adaptation to stress. Currently, it is not clear how cells could harness this process in physiological situations. However, the interplay with generation of antioxidant defences, such as GSH, with detoxifying and repairing enzymes, and with other PTMs are unveiling further possibilities for modulation of the effects of lipoxidation. Detailed knowledge of these processes will be necessary to understand its involvement in pathophysiology as well as the possibilities for therapeutic intervention.

## Figures and Tables

**Table 3:** Multiple modification mapping studies in vitro.

Protein	Targeted Residue (Position)	Electrophile	Type of Adduction	Reference
Pyruvate kinase	Cys 49, 152, 326, 358, 423, 474	Acrolein, HHE and MDA	Michael, Schiff’s or FDP adduction	[33]
	Lys 66, 115, 135, 166, 188, 207, 224, 247, 270, 305, 367, 393, 475			
	His 379, 391, 464			
Cyclin-dependent Kinase 2	Cys 177	HNE	Michael	[85]
	Lys 129			
	His 60, 71, 161, 268, 283, 295			
Serum Albumin	Cys 53, 62, 75, 101, 124, 245, 246, 253, 269, 270, 277, 514	HNE and MDA	Michael and Schiff’s (N-propenal-lysine adduct with MDA)	[137,138]
	Lys 73, 106, 136, 174, 233, 240, 281, 378, 525, 541, 545			
	His 67, 105, 128, 242, 247, 510			
Apolipoprotein E	Lys 64, 67, 68, 135, 138, 149, 155, 254	Acrolein	Michael and Schiff’s	[139]
Creatine kinase	Cys 141, 145, 254, 283	HNE	Michael and Schiff’s	[140]
	Lys 86, 101			
	His 7, 26, 29, 66, 97, 191, 219, 234, 276, 296, 305			

## Abbreviations

ACR	Alkenal/one reductase
ADH	Alcohol dehydrogenase
AER	Alkenal reductase
AKR	Aldo-keto reductase
AKR1B1	Aldo-ketoreductase B1
ALDH	Aldehyde dehydrogenase
ALDH2	Aldehyde dehydrogenase 2
ALEs	Advanced lipoxidation end products
ALH	Alkenal hydrogenase
ALR	Aldose/aldehyde reductase
CaMKII	Ca^2+^/calmodulin-dependent protein kinase II
COX	Cyclooxygenases
CRM1	Chromosomal maintenance 1
cyPG	Cyclopentenone prostaglandin(s)
CYP450	Cytochrome P450
FDP	Nε-(3-formyl-3,4-dehydropiperidino)
GAPDH	Glyceraldehyde-3-phosphate dehydrogenase
GFAP	Glial fibrillary acidic protein
GO	Gene ontology
GSH	Reduced glutathione
GST	Glutathione S-transferase
HDACs	Histone deacetylases
HNE	4-hydroxynonenal
Hsp70	Heat shock protein 70
Hsp90	Heat shock protein 90
iNOS	Nitric oxide synthase
LOX	Lipoxygenases
MDA	Malondialdehyde
NF-κB	Nuclear factor-κB
NO_2_-FA	Nitrate fatty acid
NO_2_-POPC	Nitrated phospholipid 1-palmitoyl-2-oleyl-phosphatidylcholine
Nrf2	Nuclear factor erythroid 2–related factor 2
ONE	4-oxononenal
oxo-ODE	Oxooctadecadienoic acid
PG	Prostaglandin(s)
PGA_1_	Prostaglandin A1
PGA_2_	Prostaglandin A2
PGD	Prostaglandin D
PGE_2_	Prostaglandin E2
PI3K	Phosphoinositide 3-kinase
PKA	Protein kinase A
PKC	Protein kinase C
PKG	Protein kinase G
PP1	Protein phosphatase 1
PP2A	Protein phosphatase 2
PP2B	Protein phosphatase 2B
PPARγ	Peroxisome proliferator-activated receptor γ
Prx	Peroxiredoxin
PTEN	Phosphatidylinositol 3,4,5-trisphosphate 3-phosphatase
PTM	Post-translational modification
PUFAs	Polyunsaturated fatty acids
RAGE	Receptor of advanced glycation end products
RNS	Reactive nitrogen species
ROS	Reactive oxygen species
sEH	soluble epoxide hydrolase
SOD	Superoxide dismutase
STAT3	Signal transducer and activator of transcription 3
TCA	Tricarboxylic acid cycle
Trx	Thioredoxins
UPR	Unfolded protein response
15d-PGJ_2_	15-deoxy-Δ^12,14^-prostaglandin J_2_
